# Sarcopenia and mortality in Indigenous and non-Indigenous New Zealand octogenarians—the LiLACS NZ cohort study

**DOI:** 10.1007/s41999-025-01261-5

**Published:** 2025-06-28

**Authors:** Simon A. Moyes, Vanessa Selak, Lindsay Plank, Joanna Hikaka, Ngaire Kerse

**Affiliations:** 1 Faculty of Medical and Health Sciences, Department of General Practice & Primary Healthcare, School of Population Health, University of Auckland, PO Box 92019, Auckland, 1142 New Zealand; 2 Faculty of Medical and Health Sciences, Department of Epidemiology & Biostatistics, School of Population Health, University of Auckland, Auckland, New Zealand; 3 Faculty of Medical and Health Sciences, Department of Surgery, University of Auckland, Auckland, New Zealand; 4 Waitemata District Health Board, Auckland, New Zealand; 5 Te Kupenga Hauora Māori, Faculty of Medical and Health Sciences, University of Auckland, Auckland, New Zealand

**Keywords:** Muscle strength, Sarcopenia, Mortality, New Zealand, Indigenous

## Abstract

**Purpose:**

As the population is ageing, age-related vulnerability to chronic infirmity has become of greater concern. Sarcopenia, low muscle strength and mass, is a major contributor to infirmity. Both sarcopenia and hand grip strength (HGS) are known to predict mortality in older people. This paper investigates the impact of ethnicity on this relationship in New Zealand octogenarians.

**Methods:**

This study used data from Life and Living in Advanced Age: A Cohort Study in New Zealand (LiLACS NZ), which recruited 421 Māori and 516 non-Māori with follow-up mortality data for over twelve years and had a median survival period of 6.02 years, 251 Māori and 374 non-Māori had sufficient data to be included in modelling. Survival analysis (Cox regression) assessed the association between HGS or probable sarcopenia (defined using HGS) and mortality, separately by ethnicity (Māori, non-Māori) and sex.

**Results:**

Hand grip strength was a predictor of mortality for non-Māori men (age-adjusted hazard ratio, aHR, per 1 kg increase in HGS, 0.93, 95% CI: 0.91–0.96) but not for women or Māori men. Probable sarcopenia was associated with an increased hazard of mortality among all groups, but the effect was statistically significant for non-Māori (men 1.97, 1.39–2.79; women 1.49, 1.08–2.06) and not Māori (men 1.55, 0.97–2.48; women 1.36, 0.79–2.34).

**Conclusion:**

Hand grip strength, either as a continuous variable or to identify probable sarcopenia, remains an important indicator with high clinical utility in advanced age. However, its utility may need to be re-examined for Indigenous populations.

## Key summary points

**Aim:**

To investigate the relationship of mortality to sarcopenia and hand grip strength in New Zealand octogenarians.

**Findings:**

Hand grip strength was a predictor of mortality for non-Māori men but not for women or Māori men. Probable sarcopenia was associated with an increased hazard of mortality among all groups; the effect was statistically significant for non-Māori men and women but not Māori men and women.

**Message:**

Hand grip strength, either as a continuous variable or to identify probable sarcopenia, remains an important indicator with high clinical utility in advanced age

## Introduction

### Background/rationale

New Zealand’s population is ageing, with health issues for those of advanced age seldom studied. Older people are susceptible to chronic ailments and have a high mortality rate [1]. Sarcopenia, age-related low muscle strength and mass, is an important chronic condition. Sarcopenia predicts future health outcomes [2], including mortality in older age groups [3–6]. Hand grip strength (HGS) also predicts mortality [7–17].

The Second European Working Group on Sarcopenia in Older People (EWGSOP2) proposed using HGS rather than muscle mass to screen for probable sarcopenia [2]. This change would expedite sarcopenia diagnoses in clinical practice, as muscle mass and quality are difficult to measure precisely.

The EWGSOP2 suggested HGS cut-offs for probable sarcopenia referencing British norms [18]. Subsequent studies found differences between the mean peak HGS of various ethnic groups and these British data [19–21]. Others noted that Indigenous people have lower mean HGS than the general population of their respective countries [22–24]. Most survival analyses exploring HGS or sarcopenia looked at those in their sixties and seventies with a follow-up period of less than six years [3, 8, 13]. Some studies of HGS and mortality included ethnic minorities [12, 16] but not Indigenous populations. Further research is needed among Indigenous people to establish whether HGS is associated with health outcomes for them, enabling the development of ethnic-specific strategies.

### Objectives

This paper examines the utility of HGS and probable sarcopenia in predicting mortality for Māori and non-Māori octogenarians in New Zealand.

## Methods

### Study design

The data analysed here are from LiLACS NZ, a University of Auckland cohort study that followed non-Māori from their eighty-fifth year and Māori in their eighties [1].

### Setting

The LiLACS NZ study recruited participants from the general population in one region of New Zealand between 2010 and 2011. There were six annual waves of interviews and physical examinations from 2010 to 2016. Data were usually collected at the participant’s home or a dedicated assessment centre, occasionally at a primary healthcare clinic.

Permission for researchers’ access to routinely collected Ministry of Health (MoH) data was optional in the written informed consent. These data include admission and discharge dates, any diagnoses and procedures for all publicly funded hospitalisations in the country, and the date and cause of death supplied by Births, Deaths and Marriages (BDM). As the MoH and BDM routinely collect these data independently of the study, the hospitalisation and mortality data described here extends to the middle of 2024.

### Participants

All those living in the study area, either born in 1925 or self-identified as Māori and born 1920–1930, were eligible to participate in the LiLACS NZ study. The widened age range for Māori enlarged the population pool for the Indigenous participants to achieve a similar sample size to the non-Māori group [1]. The recruitment birth year ranges were chosen to have roughly 10% mortality per annum from Statistics New Zealand’s life tables [25]. The life tables indicated non-Māori men and women aged 85 years, Māori men aged 78 years and Māori women aged 81 years had 10% mortality per annum. The Māori cohort was 80–90 years at recruitment; as their average age was likely higher than 81 years, a higher mortality rate than non-Māori was expected.

Seven local primary healthcare and Māori providers undertook recruitment, interviews and physical assessments [1]. Multiple strategies were used to identify and invite participants. Each year after recruitment, a participant would be telephoned to arrange a follow-up interview and physical assessment unless they had previously declined to participate further or died.

### Data sources/measurement

#### Outcome

If the participant died before 30th June 2024, their survival time was between the date of their initial LiLACS NZ assessment and their date of death. Those living beyond 30th June 2024 were classed as censored, and their survival time was between their initial LiLACS NZ assessment date and 30th June 2024.

#### Predictors

Muscle strength was assessed at baseline using HGS in the LiLACS NZ study as part of the physical assessment. The participant squeezed the handgrip of the Takei GRIP-D dynamometer (Takei Scientific Instruments Co., Ltd, Tokyo, Japan) whilst standing with their elbow at full extension and their arm at their side three times with each hand. The HGS estimate was the highest of the six readings. Participants who had trouble standing did the test by sitting with their arms at their side. Probable sarcopenia derived from HGS was also tested using cut-offs for probable sarcopenia from the EWGSOP2; less than 27 kg for men and 16 kg for women [2].

Participants’ age at their initial interview was controlled for in all models. Sex and ethnic grouping were self-defined in the initial interview. Serious illnesses and chronic conditions could be confounders, but these were observed too rarely to be used singly. Instead, the multimorbidity measure (M3) score was tested as a covariate. The M3 score is derived from hospitalisation data, and its development team found that in New Zealand, the M3 outperformed the Charlson and Elixhauser indices in predicting mortality [26]. The M3’s creators recommend using either a one or five-year history of hospitalisations; as LiLACS NZ had a relatively small sample size, the five years before the participant’s initial interview was selected. Smoking and body mass index (BMI) were evaluated as covariates as they could have been confounding factors.

### Bias

The LiLACS NZ team attempted to recruit as many people eligible for the study as practicable within the survey area [27]. The impact of participants’ missing data on modelling is explored below.

### Study size

A sample size of approximately 500 Māori and 500 non-Māori was chosen to detect a difference in mortality rate between different levels of nutrition risk or activities of daily living, with an expected overall mortality rate of 10% per annum [1].

### Statistical methods

All analyses were performed using SAS® software, version 9.4 (TS1M5) for Windows (SAS Institute Inc., Cary, NC).

Descriptive statistics report HGS and the proportion of the cohorts with probable sarcopenia in four subgroups based on ethnicity (Māori, non-Māori) and sex. Cox proportional hazard models assessed the effects of HGS or probable sarcopenia and the M3 comorbidity score on mortality of the four subgroups separately. Men have much higher HGS but lower survival than women, so separate models for each sex avoided the need for interaction terms in a single model. The relationship between HGS and mortality could differ between Māori and non-Māori; Māori had lower life expectancy, so separate models for each ethnicity avoided the need for interaction terms. Age was included as a covariate in all adjusted models.

Kaplan–Meier curves were graphed for the eight combinations of sex, ethnicity and sarcopenia (Men/Women, Māori/non-Māori, probable sarcopenia/not) to illustrate the age-adjusted survival of each group over time. These curves track each group’s modelled survival probability at each time point.

Absolute event rates were reported as observed differences in mortality in the first five years of follow-up. Differences in mortality at five years between predictors were assessed using the Cochran–Mantel–Haenszel test.

#### Missing data

Age, sex, and ethnicity were known for all participants, and the date of death or current survival was known for almost all. Some participants in the study did not have an initial physical assessment, so they did not have an initial HGS measurement. A few that had a physical assessment did not attempt an HGS measurement. Models classing participants as having, not having or unknown probable sarcopenia estimated the impact of these missing observations.

## Results

### Participants

The study recruited 56% of eligible Māori and 59% of eligible non-Māori in the recruitment area [28]. Both cohorts were close to the most recent census population of the same age in the proportion of women and former smokers. Still, residential care residents could have been underrepresented[27].

Participants dropped out of the study because of preference, re-locating out of the study area, illness or death; the details have been published previously [27]. Withdrawal from further participation would have only affected data collection by the MoH if the participant moved overseas, and this did not occur.

### Descriptive data

Women were 54%, and Māori 40%, of participants included in modelling (337/625, 250/625, respectively). The mean age at recruitment of Māori was 83, younger than the non-Māori mean of 85 years. Men had greater mean HGS at recruitment than women (30.4 kg (SD 6.4 kg) versus 19.1 kg (SD 4.9 kg), respectively). Mean HGS in Māori was slightly higher than in non-Māori for men and women. However, the age-adjusted HGS for Māori women was 18.5 kg, and for men it was 30.1 kg, very close to the mean for non-Māori. Most study participants had a recent history of ill health; 53% had been hospitalised with some morbidity in the five years before the study. Overall, a quarter of those tested had probable sarcopenia (Table 1). The difference in probable sarcopenia rate between Māori and non-Māori women was not statistically significant (tested by logistic regression, the age-adjusted *p*-value was 0.24).

**Table 1:** Baseline demographics for the LiLACS NZ cohorts

	Māori women		Māori men		Non-Māori women		Non-Māori men	
	*n*	Mean (SD)	*n*	Mean (SD)	*n*	Mean (SD)	*n*	Mean (SD)
Age (years)	146	82.4 (2.6)	105	81.9 (2.6)	192	84.6 (0.5)	182	84.6 (0.5)
Ethnicity^a^								
Māori	146	146 (100%)	105	105 (100%)	192	0 (0%)	182	0 (0%)
European	146	74 (50.7%)	105	38 (36.2%)	192	191 (99.5%)	182	181 (99.4%)
Pacific	146	2 (1.4%)	105	1 (1.0%)	192	1 (0.5%)	182	0 (0%)
Asian	146	1 (0.7%)	105	2 (1.9%)	192	1 (0.5%)	182	0 (0%)
Other	146	0 (0%)	105	0 (0%)	192	0 (0%)	182	1 (0.6%)
Education *n* (%) Secondary school qualification or higher	144	49 (34.0%)	103	33 (32.0%)	190	92 (48.4%)	182	91 (50.0%)
BMI (kg/m^2^)	143	29.0 (5.6)	103	30.2 (5.2)	191	26.9 (4.3)	181	26.8 (3.8)
HGS (kg)	146	19.8 (5.2)	105	30.8 (6.9)	192	18.5 (4.6)	182	30.2 (6.2)
HGS Media [Quartile 1, Quartile 3]	146	19 [17,23]	105	30 [27,34]	192	19 [15,22]	182	31 [26,34]
Probable sarcopenia	146	27 (18.5%)	105	24 (22.9%)	192	53 (27.6%)	182	52 (28.6%)
No sarcopenia	146	119 (81.5%)	105	81 (77.1%)	192	139 (72.4%)	182	130 (71.4%)
M3 morbidity Score	137	0.3 (0.5)	99	0.6 (0.8)	187	0.3 (0.5)	181	0.3 (0.6)
Without morbidity *n* (%)^b^	137	81 (59.1%)	99	39 (39.4%)	187	98 (52.4%)	181	103 (56.9%)
Number of medications	136	5.1 (3.3)	94	5.1 (3.4)	186	5.5 (3.3)	172	5.2 (3.6)
Not on any meds *n* (%)	136	12 (8.8%)	94	8 (8.5%)	186	9 (4.8%)	172	8 (4.7%)
3MS (/100)	159	87.5 (15.1)	107	84.3 (16.3)	209	92.2 (10.0)	184	91.0 (10.8)
3MS < 70/100 *n* (%)	159	10 (6%)	107	11 (10%)	209	6 (3%)	184	6 (3%)
Smoking *n* (%)								
Never	143	76 (53.1%)	105	31 (29.5%)	192	132 (68.8%)	182	62 (34.1%)
Current	143	19 (13.3%)	105	11 (10.5%)	192	7 (3.6%)	182	12 (6.6%)
Past	143	48 (33.6%)	105	63 (60.0%)	192	53 (27.6%)	182	108 (59.3%)

*N*, total number of LiLACS NZ participants

Age, age at time of first assessment (years)

BMI, Body Mass Index (kg/m^2^)

HGS, Hand Grip Strength (kg of force)

M3, a comorbidity scale with a score of 0 being no comorbidities. Applied to the previous five years hospitalisations

3MS, the Modified Mini-Mental State Examination a hundred-point scale of cognition with a score of 100 being the highest cognition and 0 being unable to answer any question correctly

^a^Self-identified, more than one could be selected

^b^M3, the multimorbidity measure score of zero

Only two-thirds of participants felt willing and able to be physically assessed, resulting in 60% of Māori and 72% of non-Māori having their HGS measured (251/421 and 374/516, respectively). The M3 score was calculable for those who granted permission to access their MoH records, 90% of Māori and 97% of non-Māori (379/421 and 498/516, respectively). The date of death or assurance of continued survival was available for 99% of Māori and non-Māori due to MoH/BDM records, newspaper obituaries and attempts at contact by study researchers (417/421 and 515/516, respectively).

### Outcome data

Participants still living at the time of analysis had survived more than twelve years since their initial assessment. The median survival period for the entire cohort was 6.02 years (IQR 2.96–9.79), and 881 (94%) of the study participants died in the follow-up period (2010 to 30th June 2024).

### Main results

**Fig. 1:**
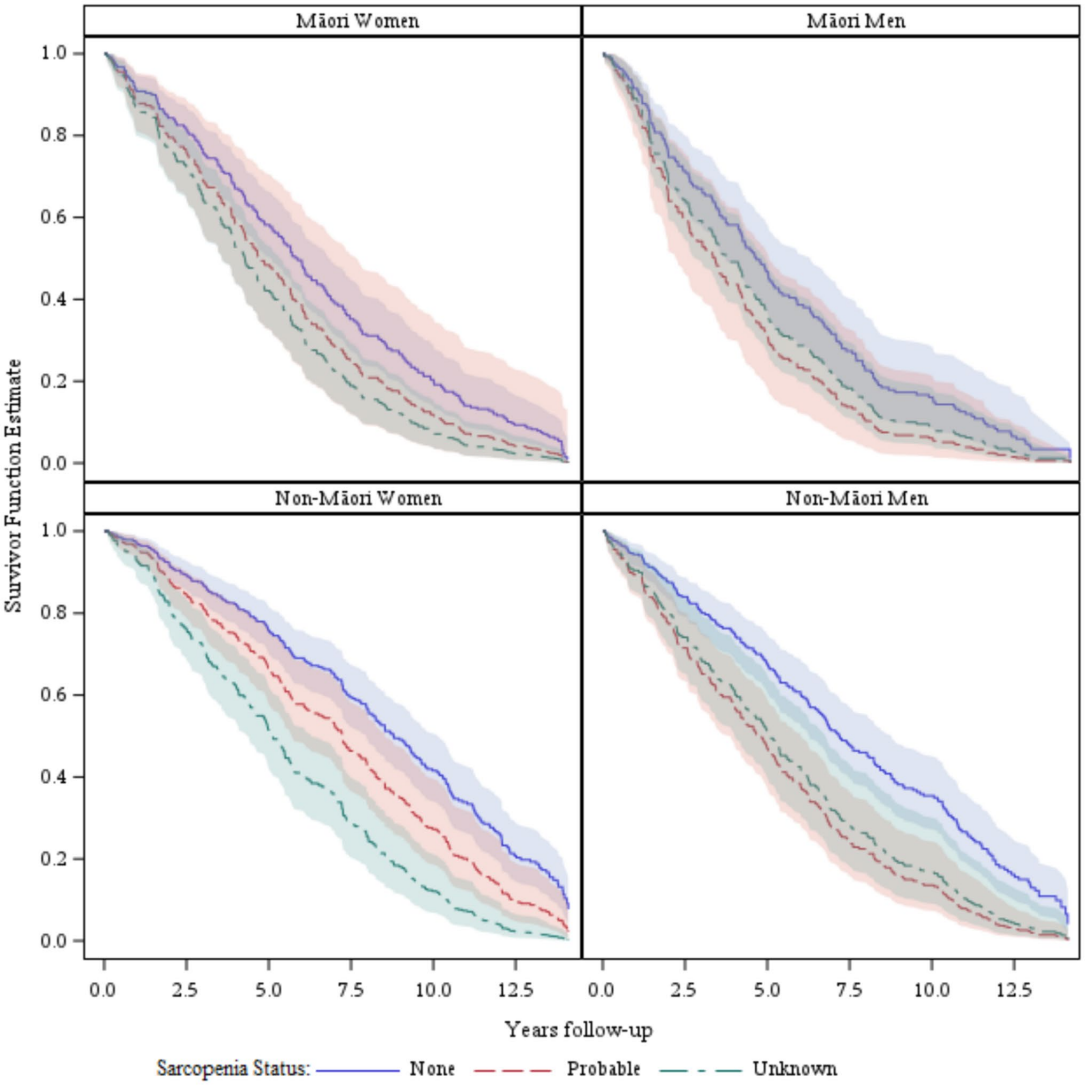
Age-adjusted Kaplan–Meier curves of the survival of LiLACS NZ participants in the ten years from joining the study by sex, ethnicity and sarcopenia status

There were significant differences in mortality after five years of follow-up between the sexes (48% of men, 37% of women, *p*-value = 0.0009), Māori and non-Māori (47% and 38%, respectively, *p*-value 0.005), and between Māori born in 1920 and in 1930 (90% and 35%, respectively, p-value 0.0009). Mortality was higher for those with probable sarcopenia than without (48% and 28%, respectively, *p*-value < 0.0001). Adjusted for age, the two groups with better survival were non-Māori women with or without probable sarcopenia (Fig. 1).

Separate age-adjusted models were constructed for Māori and non-Māori, men and women, as those who were older, Māori and men, were at significantly higher risk of death (Table 2). Having greater HGS was a significant predictor of survival for non-Māori men but not non-Māori women. Grip strength was not a significant predictor of survival for either Māori men or women.

**Table 2:** Cox proportional hazard models for the all-cause mortality of LiLACS NZ participants using hand grip strength (HGS) as a predictor

	Unadjusted model		Age-adjusted model		Morbidity adjusted model	
	Hazard ratio (95% CI)	*p*-value	Hazard ratio (95% CI)	*p*-value	Hazard ratio (95% CI)	*p*-value
Māori women						
Age (+1 year)			1.16 (1.09, 1.23)	< 0.0001	1.16 (1.09, 1.24)	< 0.0001
Grip strength (+1 kg)	0.97 (0.93, 1.01)	0.10	0.99 (0.95, 1.03)	0.50	1.00 (0.96, 1.04)	0.84
Comorbidity Score (+1 point)					1.69 (1.35, 2.11)	< 0.0001
Māori men						
Age (+1 year)			1.02 (0.94, 1.11)	0.63	1.04 (0.95, 1.13)	0.38
Grip strength (+1 kg)	0.98 (0.95, 1.01)	0.12	0.98 (0.95, 1.01)	0.12	0.98 (0.95, 1.01)	0.22
Comorbidity Score (+1 point)					1.47 (1.09, 1.97)	0.011
Non-Māori women						
Age (+1 year)			1.07 (0.80, 1.42)	0.66	1.09 (0.82, 1.45)	0.56
Grip strength (+1 kg)	0.98 (0.95, 1.02)	0.33	0.99 (0.95, 1.02)	0.37	0.98 (0.95, 1.01)	0.26
Comorbidity Score (+1 point)					1.54 (1.25, 1.88)	< .0001
Non-Māori men						
Age (+1 year)			0.87 (0.65, 1.15)	0.33	0.83 (0.63, 1.11)	0.22
Grip strength (+1 kg)	0.93 (0.91, 0.96)	< 0.0001	0.93 (0.91, 0.96)	< 0.0001	0.94 (0.91, 0.97)	< 0.0001
Comorbidity Score (+1 point)					1.65 (1.31, 2.07)	< 0.0001

For effective sample size, death rate and average length of follow-up in these models see Table 4

Probable sarcopenia (defined by the HGS cut-offs) was a significant predictor of mortality for non-Māori men and women (Table 3). Probable sarcopenia was not a significant predictor of survival for Māori men or women.

**Table 3:** Cox proportional hazard models for the all-cause mortality of LiLACS NZ participants using probable sarcopenia as a predictor

	Unadjusted model		Age-adjusted model		Morbidity adjusted model	
	Hazard ratio (95% CI)	*p*-value	Hazard ratio (95% CI)	*p*-value	Hazard ratio (95% CI)	*p*-value
Māori women						
Age (+1 year)			1.17 (1.09, 1.25)	< 0.0001	1.16 (1.09, 1.25)	< 0.0001
Sarcopenia (probable vs none)	1.39 (0.89, 2.19)	0.15	1.36 (0.79, 2.34)	0.28	1.19 (0.69, 2.06)	0.53
Comorbidity Score (+1 point)					1.67 (1.33, 2.09)	< 0.0001
Māori men						
Age (+1 year)			1.02 (0.94, 1.11)	0.64	1.04 (0.95, 1.14)	0.40
Sarcopenia (probable vs none)	1.55 (0.97, 2.48)	0.067	1.55 (0.97, 2.48)	0.066	1.51 (0.98, 2.33)	0.062
Comorbidity Score (+1 point)					1.51 (1.14, 2.00)	0.0045
Non-Māori women						
Age (+1 year)			1.05 (0.79, 1.39)	0.75	1.07 (0.81, 1.41)	0.65
Sarcopenia (probable vs none)	1.50 (1.08, 2.08)	0.015	1.49 (1.08, 2.06)	0.016	1.48 (1.07, 2.05)	0.017
Comorbidity Score (+1 point)					1.52 (1.25, 1.86)	< 0.0001
Non-Māori men						
Age (+1 year)			0.90 (0.68, 1.19)	0.46	0.88 (0.66, 1.16)	0.36
Sarcopenia (probable vs none)	1.94 (1.39, 2.71)	0.0001	1.97 (1.39, 2.79)	0.0001	1.75 (1.23, 2.48)	0.0018
Comorbidity Score (+1 point)					1.64 (1.28, 2.10)	< 0.0001

For effective sample size, death rate and average length of follow-up in these models see Table 4

### Other analyses

Probable sarcopenia remained significant when the M3 comorbidity score was added to the models for non-Māori men and women (Table 3). In those models, the M3 score was a highly significant predictor of mortality for Māori and non-Māori men and women. Body mass index was not a significant predictor of mortality in these models, and the significance of grip strength and sarcopenia remained unchanged. Smoking status was a significant predictor of mortality for Māori women and non-Māori men, but not Māori men or non-Māori women. In all models, the significance of grip strength and sarcopenia remained unchanged.

‘Unknown’ was added as a third category to the sarcopenia classification for people whose HGS was not recorded. The three-category variable was a highly significant predictor of mortality for Māori women, non-Māori women and men (*p*-values of 0.0095, < 0.0001 and < 0.0001, respectively). In these three groups, those with missing HGS data had significantly lower survival than those with normal HGS (Fig. 1).

## Discussion

### Key results

Māori men and women had a higher mean HGS but a shorter survival period on average. The shorter life expectancy for Māori participants was predictable from Statistics New Zealand’s life tables [25]. The greater HGS of Māori participants on average was due to age difference; the age-adjusted HGS for Māori was almost exactly that observed for non-Māori. The HGS means for men and women in this study were close to those observed in Europe and higher than for this age group globally [19]. Māori having age-adjusted mean HGS essentially identical to non-Māori is novel; in other countries, minority Indigenous people have lower mean HGS than their general population [22–24]. Lower HGS was an indicator of greater mortality risk in non-Māori men. The underlying reasons why HGS was not a significant predictor of mortality for Māori could not be established, and further research would be needed to identify all of them.

The association between HGS and mortality for both Māori and non-Māori was similar to the Newcastle 85+ cohort study of 85-year-olds in Britain, which reported an HR for mortality for men of 0.97 (0.95–0.99) and women of 0.96 (0.94–0.99) favouring higher HGS [14].

The HGS cut-offs of 27 kg for men and 16 kg for women identified a group with probable sarcopenia that had significantly higher mortality among non-Māori men and women. The association between probable sarcopenia and mortality for non-Māori men was stronger than that reported in a meta-analysis (pooled HR 1.60, 95%CI 1.24–2.06) [3]. The magnitude of the association between probable sarcopenia and mortality was similar for non-Māori women and Māori men and women; however, the sample size of survival models was only large enough (Table 4) to achieve statistical significance for non-Māori women.

**Table 4:** Sample sizes of survival models

	Unadjusted/age-adjusted models	Morbidity adjusted model	Including unknown status model
Māori women			
Died/Total (%)	129/145 (89%)	123/137 (90%)	217/241 (90%)
Mean follow-up (yrs.)	7.77	7.90	6.66
Māori men			
Died/Total (%)	102/105 (97%)	96/99 (97%)	171/176 (97%)
Mean follow-up (yrs.)	5.59	5.65	5.18
Non-Māori women			
Died/Total (%)	176/192 (92%)	172/187 (92%)	260/278 (94%)
Mean follow-up (yrs.)	8.15	8.22	7.26
Non-Māori men			
Died/Total (%)	175/182 (96%)	174/181 (96%)	228/237 (96%)
Mean follow-up (yrs.)	6.72	6.73	6.33

The lower proportion of Māori women with probable sarcopenia could reflect a lower rate of sarcopenia in the population. Further study would be needed to confirm this and establish if lifestyle factors like diet, exercise or cultural practices have been beneficial.

The M3 comorbidity score predicted mortality in Māori and non-Māori men and women. However, adding M3 to the models, sarcopenia remained an independent predictor of mortality.

Māori women, non-Māori men and women who did not perform the HGS test had significantly higher mortality than those with normal HGS. These results suggest that poor health could have influenced the participant not to attempt the HGS test. Despite this, the HGS is a much easier, physically and mentally, test for older people and can have higher compliance than other standard physical tests [29].

Grip strength did predict mortality in New Zealand non-Māori octogenarians but was not a significant predictor for Māori. The aHRs for Māori were like the non-Māori, but the relationship between sarcopenia and mortality remains unproven for Māori. The utility of muscle strength as a predictor of health outcomes for Māori requires further examination.

### Limitations

#### Data source

Proportionately, fewer aged care facility residents were in the study than were in the general population [27]. The LiLACS NZ researchers tried to accommodate those already in poor health [28], but people still declined to participate because of their poor health. The outcome of the missing data model indicates that a disproportionate number of those who felt well enough to participate in the study did not feel well enough to have their HGS assessed.

The M3 score relies on hospital data; if a study participant did not visit a hospital during the period used for calculating the M3, they would be scored as having no comorbidities. Underreporting could have been a greater problem for the Māori cohort due to inequities in access to hospital services by Māori, particularly those living rurally [30, 31]. However, over 95% of Māori and non-Māori men and women permitting access to their records visited a hospital during the follow-up period. Māori men had the highest mean M3 score and were the only group where the majority had comorbidities indicated.

#### Outcome

The drawback of all-cause mortality is that deaths can result from accidents or such rapid deterioration of health that they cannot be predicted from health assessments done a year or more earlier. Over ten years, there was a risk for study participants to develop comorbidities they did not have at baseline. Future research could use repeated HGS measurements, with M3 scores recalculated for each assessment, to evaluate the impact of changes in HGS and the number of comorbidities on longevity.

#### Hand grip strength

There is no universally agreed method of measuring HGS; slightly higher HGS readings are observed with the test subjects standing with their arm at their side than when they were tested sitting down with their elbow at 90° [32]. Readings can vary between equipment from different manufacturers [33]. The EWGSOP2 cut-offs came from a paper that pooled data from twelve studies; most used the JAMAR dynamometer while seated, only the Newcastle 85+ used the same Takei device while standing [18]. The authors checked but did not find adjusting for the differing methods and devices necessary when pooling the data in their paper. As LiLACS NZ used the same dynamometer and methods with the same age group as Newcastle 85+, so results should have a similar level of compatibility with other studies.

#### Probable sarcopenia

The EWGSOP2 team acknowledged that their selection of cut-offs for HGS and other measures was arbitrary and a topic of further research. The EWGSOP2 procedure for defining sarcopenia identifies probable sarcopenia using muscle strength. Then, it confirms the diagnosis by determining poor muscle mass through a body composition scan or bioelectrical impedance analysis [2]. There are other reasons for low strength than sarcopenia, such as degraded neuromuscular signals [34]. However, in the LiLACS NZ study, few participants had dementia, and excluding those with a Modified Mini-Mental State Examination (3MS) below 70 (Table 1) had a negligible effect on the models.

### Interpretation

Previous studies noted that HGS [7–17] and sarcopenia predict mortality [3–6]. Age, sex and ethnicity were strong predictors of mortality in LiLACS NZ and need to be considered as factors in survival. Non-Māori women without probable sarcopenia had the best survival rate, with non-Māori women with probable sarcopenia having the next best. For non-Māori men, HGS and probable sarcopenia were highly significant in predicting mortality. Classifying non-Māori women as having probable sarcopenia or not using HGS was a significant predictor of mortality despite the continuous measure not being significant for this group. Neither HGS nor probable sarcopenia predicted mortality significantly for Māori men or women. The estimated aHRs for sarcopenia for Māori men, women and non-Māori women were similar. The Māori aHRs might also have shown a significant relationship between HGS and mortality had their sample size been as big as for non-Māori women (Tables 3, 4). However, the overall high mortality of Māori men meant the difference in mortality rate between the strongest and weakest members of the cohort was considerably less than the other groups (Māori men’s mortality at five years was 53% with probable sarcopenia and 46% for those without; non-Māori men 53% and 30%, respectively). The low survival rate of Māori women, non-Māori men and women whose sarcopenia status was unknown indicated that those unable to be tested using HGS could have had higher mortality than those who tested poorly. The highest participation in HGS testing and the most significant relationship between HGS and mortality was non-Māori men (Table 2). The results presented here support other research calling for ethnic-specific considerations of health indices and question the utility of muscle strength as a predictor of health outcomes for Māori in New Zealand [2].

### Generalisability

The results here indicate probable sarcopenia diagnosed using internationally recognised HGS cut-offs was predictive of mortality in the non-Māori cohort. Sarcopenia had a similar association with mortality for both the Māori and non-Māori cohorts. However, the Māori sample size was smaller, and the survival of those with high HGS was lower for Māori than non-Māori. Early detection of sarcopenia would initiate any course of treatment while still feasible. Previous studies have identified various possible treatments for sarcopenia, including exercise, dietary supplements and even electrical stimulation[35–37]. Further research will be needed to gauge the utility of HGS as a screening tool for targeting treatments. The results suggest that treatment for sarcopenia would have a higher impact on non-Māori than Māori mortality.

## Data Availability

The data supporting this study’s findings are not openly available due to the permissions granted to the study investigators by the study participants. They are available from the corresponding author upon reasonable request. Data are located in secure data storage at the University of Auckland.
